# Pulmonary Tuberculosis in Humanized Mice Infected with HIV-1

**DOI:** 10.1038/srep21522

**Published:** 2016-02-24

**Authors:** Rebecca J. Nusbaum, Veronica E. Calderon, Matthew B. Huante, Putri Sutjita, Sudhamathi Vijayakumar, Katrina L. Lancaster, Robert L. Hunter, Jeffrey K. Actor, Jeffrey D. Cirillo, Judith Aronson, Benjamin B. Gelman, Joshua G. Lisinicchia, Gustavo Valbuena, Janice J. Endsley

**Affiliations:** 1 University of Texas Medical Branch, Galveston, TX 77555, USA; 2 University of Texas El Paso, El Paso, TX 79902, USA; 3 University of Texas-Houston Health Science Center, Houston, TX 77030, USA; 4 Texas A&M Health Sciences Center, College Station, TX 77853, USA

## Abstract

Co-infection with HIV increases the morbidity and mortality associated with tuberculosis due to multiple factors including a poorly understood microbial synergy. We developed a novel small animal model of co-infection in the humanized mouse to investigate how HIV infection disrupts pulmonary containment of *Mtb*. Following dual infection, HIV-infected cells were localized to sites of *Mtb*-driven inflammation and mycobacterial replication in the lung. Consistent with disease in human subjects, we observed increased mycobacterial burden, loss of granuloma structure, and increased progression of TB disease, due to HIV co-infection. Importantly, we observed an HIV-dependent pro-inflammatory cytokine signature (IL-1β, IL-6, TNFα, and IL-8), neutrophil accumulation, and greater lung pathology in the *Mtb*-co-infected lung. These results suggest that in the early stages of acute co-infection in the humanized mouse, infection with HIV exacerbates the pro-inflammatory response to pulmonary *Mtb*, leading to poorly formed granulomas, more severe lung pathology, and increased mycobacterial burden and dissemination.

In the wake of the human immunodeficiency virus (HIV) pandemic, tuberculosis (TB) persists as a global health crisis that causes an estimated 1.5 million deaths per year^1^. TB is now the most significant health threat for those living with HIV/acquired immunodeficiency syndrome (AIDS) due to multifactorial effects of HIV infection to promote susceptibility to *Mycobacterium tuberculosis* (*Mtb)* infection or reactivation, exacerbate disease, increase drug resistance development, and compromise diagnosis^2^,^3^,^4^,^5^,^6^. Treatment of co-infected persons is further complicated by drug interaction and malabsorption issues when combining anti-retroviral agents and TB chemotherapy [reviewed^7^]. Addressing these clinical challenges to reduce TB disease requires a much greater understanding of co-infection pathophysiology to inform development of novel interventions.

The human host tropism of HIV has limited the *in vivo* study of HIV and *Mtb* co-infection in an experimentally controlled system. In human subjects, the loss of CD4^+^ T cells and functional suppression of T cells and antigen presenting cell populations during chronic HIV is well known to impair the cell-mediated immune response to *Mtb* [reviewed in^8^,^9^]. The effect of infection and CD4^+^ T cell depletion by simian immunodeficiency virus (SIV) to compromise host containment of latent TB has been established through elegant studies in cynomolgus and rhesus macaque non-human primates (NHP) co-infection models^10^,^11^. A synergistic relationship between *Mtb* and HIV, however, occurs even before the loss of CD4^+^ T cells characteristic of AIDS^6^,^12^. Limited studies of human lung and bronchiole alveolar lavage fluid from co-infected subjects suggest that a poorly organized, and potentially inflammatory, pulmonary response can also occur^13^,^14^,^15^. To date, however, the immune disturbances and pathogenesis of TB/HIV in the lung prior to peripheral T cell depletion and generalized immune suppression are poorly understood due to the paucity of available tissue samples.

New animal models to complement clinical research with human subjects and basic studies of SIV and *Mtb* in NHP are needed. The bone marrow, liver, thymus (BLT) humanized mouse (HuMouse) has facilitated pioneering studies of HIV pathogenesis and treatment^16^,^17^,^18^,^19^. We previously developed a model of TB in the BLT HuMouse^20^ that reproduces important hallmark features of human TB disease pathology, as described in recent independent reviews^21^,^22^. In the current studies we applied our HuMouse model of experimental TB to investigate pulmonary *Mtb*/HIV-1 co-infection. We chose to model one of several important clinical scenarios, that of new pulmonary *Mtb* infection in subjects with an existing HIV infection. Recent reports indicate that in HIV + subjects, *Mtb* infections are frequently a new event rather than reactivation of latent infections^23^,^24^,^25^. We observed that HIV-infected cells were localized to sites of *Mtb*-driven inflammation and mycobacterial replication in both human and HuMouse co-infected lung. We also observed increased mycobacterial burden and loss of granuloma structure due to HIV co-infection, consistent with disease in human subjects. Further, we show neutrophil infiltration and tissue necrosis due to HIV in the co-infected lung and increased production of pro-inflammatory cytokines and chemokines that recruit neutrophils. These results suggest that in the early stages of acute pulmonary co-infection, HIV infection compromises antibacterial functions in the lung and activates non-protective inflammatory responses and neutrophil influx that can promote tissue damage.

## Results

### HIV replication in the *Mtb*-infected BLT humanized mouse

**Figure 1:**
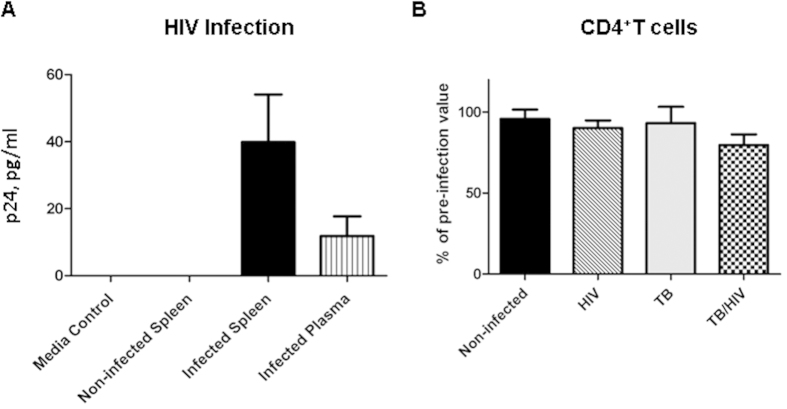
HIV infection in HuMice co-infected with *Mtb*. BLT HuMice were infected i.v. with 2,500 TCID_50_ of HIV-1 (JR-CSF) or mock infected (PBS). Subsets of HuMice from HIV or non-infected mice were infected i.n. with 250 CFU *Mtb* (H37Rv), 3 wk post-HIV infection. Data are means ± SEM from samples of non-infected control (n = 4) and three infection groups, HIV (n = 3), *Mtb* (n = 6) and *Mtb*/HIV (n = 8), of HuMice. (**A**) HIV p24 capsid protein was detected in HuMouse spleen and plasma by ELISA at 8 and 5 wk p.i. with HIV, and *Mtb*, respectively. Viral load did not differ among HIV and *Mtb*/HIV infection groups and results are thus pooled. (**B**) Flow cytometric detection of human CD45^ + ^CD3^ + ^CD4^ + ^T cells in peripheral blood, shown as a percent of baseline (pre-HIV infection) value at 5 wk p.i. with *Mtb*.

To emulate human HIV infection in BLT HuMice, animals were given 2,500 TCID_50_ of the JR-CSF strain of HIV-1 i.v. as described^16^. At 3 wk p.i. with HIV, some groups of animals were infected i.n. with 250 CFU of the H37Rv strain of *Mtb* as described^20^. Replication of HIV, based on detection of the p24 capsid protein by an ELISA assay employed in clinical settings to determine HIV status, was observed in the plasma and spleen at 8 wk post-HIV infection (Fig. 1A). Expression of p24, though variable, was detectable in HIV- and HIV/*Mtb*-infected mice from two independent studies. Co-infection with *Mtb* did not significantly alter viral load at this stage of infection (5 wk post-*Mtb*, and 8 wk post-HIV, infection). As previously observed in HuMice infected with the JR-CSF strain of HIV-1, CD4^+^ T-cell numbers decline slowly^26^. The moderate decrease in CD4^+^ T-cells observed in HIV-infected animal groups did not reach significance compared to non-infected and *Mtb*-infected animals (Fig. 1B).

### HIV p24 + cells localize to *Mtb* lesions in the lung

**Figure 2:**
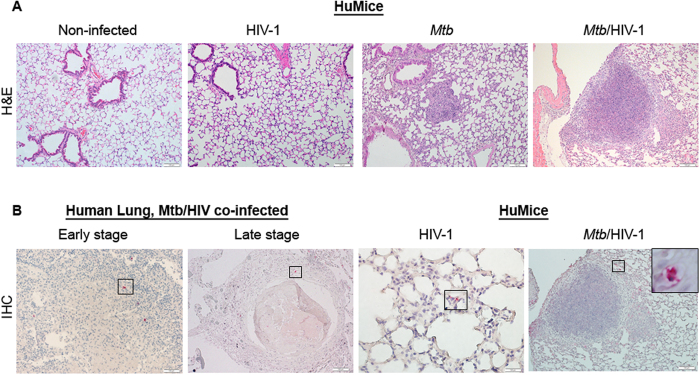
HIV-infected cells localize to *Mtb* lesions in the lung. Animals were infected i.v. with 2500 TCID_50_ HIV-1 (JR-CSF) or mock infected and 3 wk later infected i.n. with 250 CFU of *Mtb* H37Rv. (**A**) Tissue pathology visualized by H&E staining shows normal lung architecture in a non-infected and HIV-infected animals and foci of inflammation in *Mtb*- and *Mtb*/HIV-infected lung. (**B**) Detection of HIV p24 by IHC (fast red) in lung of co-infected human reference samples at early and later stages of lesion progression, and in HIV- and HIV/*Mtb*-infected HuMice 8 and 5 wk p.i. with HIV and *Mtb,* respectively.

The role of HIV-infected cells in local immune suppression at the site of *Mtb* containment in protective granulomas is an important unknown in co-infection biology. We used hematoxylin and eosin (H&E) and immunohistochemistry (IHC) to assess tissue pathology associated with the presence and distribution of HIV + cells in *Mtb*-co-infected human and HuMouse lung tissue (Fig. 2). In the absence of HIV, the lesions observed in the lung of *Mtb*-infected animals were characteristic of TB where solid lesions arise from inflammation in the interstitium in response to *Mtb*-infected macrophages as shown with representative lung in Fig. 2A. In contrast, a much greater inflammatory cell influx with involvement of alveolar spaces was observed in the lung of *Mtb*/HIV co-infected animals (Fig. 2A). Histological examination of the lung tissue from HuMice infected with only HIV revealed no remarkable pathology compared to non-infected animals (Fig. 2A).

We found that HIV + cells trafficked to the lung in HuMice infected with HIV or co-infected with HIV and *Mtb*. In the absence of *Mtb* infection, HIV p24 + cells were occasionally found and were randomly distributed in the lung parenchyma (Fig. 2B) as described^27^. In a subset of co-infected HuMice, HIV + cells were observed at sites of *Mtb* infection-driven inflammatory foci (Fig. S1A) at 3 wk p.i. with *Mtb*. Following 5 wk of co-infection, we observed that HIV + cells were localized almost exclusively to the sites of TB lesions including within developing pulmonary granulomas (Fig. 2B). The distribution of HIV + cells in co-infected HuMouse lung was similar to what we observed in co-infected human lung reference tissues. As shown with representative tissues from *Mtb*/HIV co-infected human lung from autopsy cases (Fig. 2B), we found that HIV + cells are localized primarily to the lesion periphery in both newly forming solid lesions as well as in more progressed and necrotic TB granuloma.

### Pulmonary growth of *Mtb* is increased by HIV co-infection prior to peripheral CD4^+^ T cell depletion

**Figure 3:**
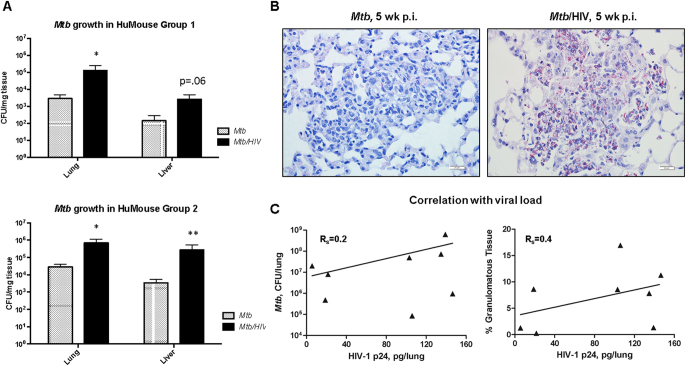
Pulmonary growth of *Mtb* is increased by HIV co-infection prior to peripheral CD4^+^ T cell depletion. (**A**) CFU enumeration of *Mtb* in lung and liver tissue of HuMice at 5 wk p.i. with *Mtb* from two independent studies. Statistically significant differences compared to non-infected are designated by *p < 0.05. (**B**) Increased bacterial growth confirmed by Ziehl-Neelson detection of AFB by light microscopy in representative lung of *Mtb*-infected (left) and *Mtb*/HIV co-infected mice (right). (**C**) Correlation between HIV p24 and mycobacterial proliferation or pathology in the lung of co-infected HuMice (n = 8) was determined using a Spearman’s rank correlation co-efficient (R_s_).

**Figure 4:**
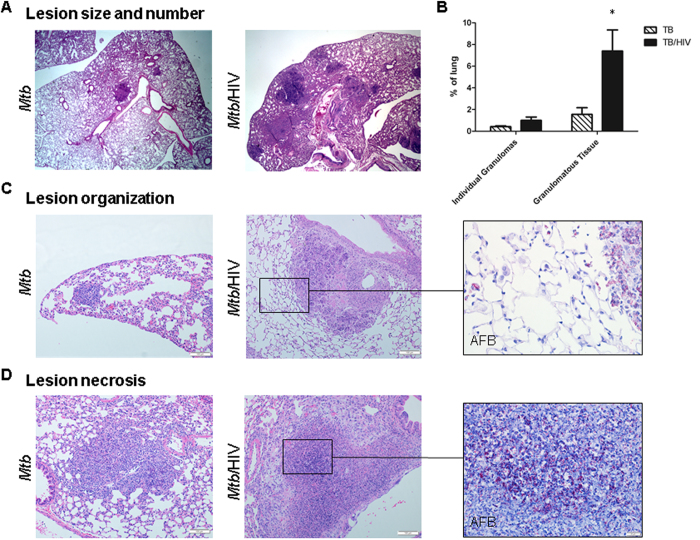
HIV co-infection exacerbates *Mtb* pathology in HuMouse lung. Determination of pulmonary pathology using H&E visualization and light microscopy. (**A**) Representative lung from HuMouse 5 wk p.i. with *Mtb* or *Mtb*/HIV demonstrating increased number and/or size of TB lesions due to HIV. (**B**) Area of the lung, shown as a %, occupied by the average individual granuloma, or total granulomatous tissue, in tissue of HuMice infected with *Mtb* (n = 6) or *Mtb*/HIV (n = 8) as determined using Image J software. (**C**) Diffuse lesion development and poor containment of *Mtb*-infected macrophages due to HIV co-infection. (**D**) Increased pulmonary pathology and bacterial burden in necrotic tissue in co-infected animals. Insets show detection of AFB using Ziehl-Neelson staining in matched tissue sections. Statistically significant differences among treatment groups are designated as follows: *p < 0.05.

To determine the impact of HIV co-infection on the growth and dissemination of *Mtb* in HuMice, we measured colony-forming units (CFU) of mycobacteria in disrupted lung and liver tissue following necropsy. Analysis of randomly selected animals at 3 wk p.i. with *Mtb* revealed moderate mycobacterial growth in the lung that was similar among animals with mock or HIV co-infection (Fig. S1B). At 5 wk p.i., however, *Mtb* load was significantly increased (>1 log) in the lung of animals co-infected with HIV (Fig. 3A) in two independent groups of animals reconstituted with tissues from different human donors. Increased bacterial load due to HIV infection was also observed in the liver, though the change did not reach significance (p = 0.06) in one experimental group. These results were verified by visualization of AFB numbers using light microscopy (Fig. 3B). As shown in Fig. 3B relative to a similarly sized TB lesion, few AFB are observable in the lesions of an *Mtb*-infected HuMouse lung while greater AFB staining is observable in the co-infected lung. To determine if there was a positive relationship between relative lung viral load and bacterial burden among the co-infected animals, a Spearman’s Correlation analysis was performed. As shown in Fig. 3C, there was a positive, though weak, relationship between HIV p24 levels and mycobacterial proliferation in lung tissue. A moderately positive relationship, however, was observed among HIV p24 and the % of granulomatous tissue in the co-infected animals (Figs 3C and 4B). These positive correlations were not statistically significant with this sample size.

### HIV co-infection exacerbates pulmonary TB pathology

A limited number of observational studies of human lung has suggested that HIV disrupts TB granuloma architecture^13^,^14^, but controlled experiments still are needed given the paucity of animal models. To determine how HIV alters TB pathology in the HuMouse, we compared lung tissue of *Mtb* and *Mtb*-HIV co-infected mice. Analysis of lung tissue from a subset of *Mtb-* or *Mtb*/HIV-infected HuMice harvested at 3 wk p.i. with *Mtb* revealed mostly non-remarkable pathology with only occasional foci of inflammation observed at sites of *Mtb* proliferation (Fig. S1). Surprisingly, a greater cellular influx was observed at these infrequent sites of *Mtb*-driven inflammation in HuMice with HIV co-infection (Fig. S1A). At this stage of disease in the HuMouse model the mycobacterial burden did not differ among HIV + and HIV- animals (Fig. S1B).

By 5 wk p.i., analysis of the gross lung demonstrated that co-infected mice had more numerous TB lesions compared to those infected with only *Mtb* (Fig. 4A). The lesion size varied among lesions within the lung, but overall the lesions in co-infected mice were larger (Fig. 4A). As a result, the percent of the lung occupied by granulomatous tissue was significantly greater in co-infected animals (Fig. 4B). These larger lesions exhibited decreased organization of the granuloma structure due to the presence of diffuse cellular aggregations at the lesion site versus the smaller and more compact lesions observed in animals infected with *Mtb* alone (Fig. 4C). Importantly, the lesions from co-infected animals had a less discriminate distribution of AFB relative to lung pathology (Fig. 4C). In the lung of animals infected with *Mtb*, AFB were fewer and were localized primarily within solid lesions (Fig. 3B), suggesting successful containment by the host immune system. In contrast, AFB + cells were observed in co-infected lung within the granulomas, but also within macrophages in the alveolar spaces adjacent to poorly defined lesion peripheral zones (Figs 3B and 4C). Increased tissue necrosis accompanied by areas of nuclear karyorrhectic debris, was also present in co-infected animals (Fig. 4D). Consistent with the reported role of necrotic tissue to promote mycobacterial growth^28^,^29^, large numbers of AFB were visualized within these necrotic tissue zones (Fig. 4D).

### Co-infection with HIV promotes neutrophil accumulation in pulmonary TB granulomas

**Figure 5:**
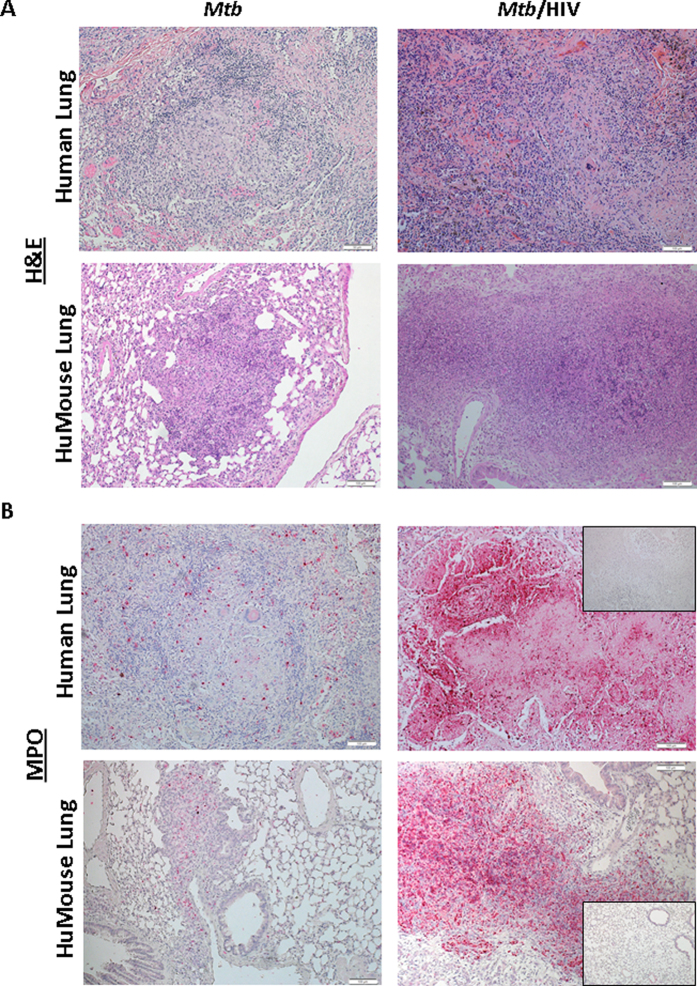
Co-infection with HIV promotes neutrophil accumulation in pulmonary TB granulomas. (**A**) Visualization of lung pathology due to *Mtb* infection and *Mtb*/HIV co-infection in reference human (top), and representative humanized mouse (bottom) lung, demonstrating diffuse lesions and tissue damage. (**B**), Determination of neutrophil accumulation due to *Mtb* or *Mtb*/HIV infection through detection of myeloperoxidase (MPO) in infected human (top) and HuMouse lung (bottom) tissue. Top inset shows non-specific control staining for fast red in the necrotic lesion. Bottom inset shows representative tissue from HuMouse with HIV mono-infection, demonstrating lack a paucity of neutrophils and non-remarkable pathology in the absence of *Mtb* co-infection.

To determine the basis for exacerbated lung pathology due to co-infection, we investigated neutrophil influx in lung tissue of HuMice and of human reference tissues. Inflammatory lesions most similar in size and stage of organization were chosen through H&E visualization of pathology for assessment alongside tissue from HuMice (Fig. 5A). Neutrophils were stained using titration of an antibody specific to myeloperoxidase (MPO) (Fig. 5B). In the HuMouse, at 5 wk pi we observed solid and well organized lesions (Figs 4C and 5A) that were similar to smaller lesions found in human lung from a decedent with *Mtb* infection (Fig. 5A). Moderate numbers of neutrophils were distributed throughout the lung lesions from HuMice and human subjects infected with *Mtb* only, consistent with prior descriptions in human lung^30^ and other animal models of TB^31^,^32^,^33^. In contrast, in co-infected lung from both human subjects and HuMice, we observed sites of inflammatory pathology characterized by a poorly organized inflammatory influx (Fig. 5A). Detection of MPO revealed a marked increase in the numbers of neutrophils localized to these poorly organized inflammatory areas in co-infected tissue (Fig. 5B). The specificity of staining in these necrotic tissues was confirmed using a non-specific primary antibody as a control (Fig. 5B, insert).

### HIV co-infection activates excessive pro-inflammatory cytokine responses to *Mtb* in the lung

**Figure 6:**
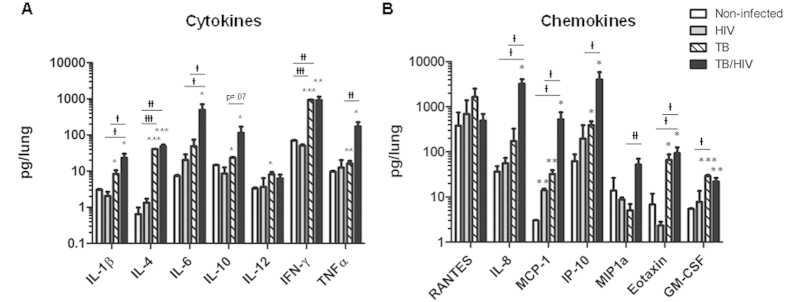
HIV co-infection activates excessive pro-inflammatory cytokine and chemokine responses to *Mtb* in the lung. Lung supernatants from HuMice harvested 5 wk p.i. with *Mtb* or *Mtb*/HIV were used to determine activation of human cytokines and chemokines by multiplex ELISA. (**A**) Expression of human cytokines, and (**B**) chemokines, in non-, HIV-, *Mtb*-, and *Mtb*/HIV-infected HuMice. Data are means ± SEM from samples of non-infected control (n = 4) and three infection groups of Humice including HIV (n = 3), *Mtb* (n = 6) and *Mtb*/HIV (n = 8). Statistically significant differences compared to non-infected controls are designated as follows: *p < 0.05; **p < 0.01; ***p < 0.001. Significant differences among infected groups are shown as 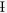
p < 0.05; 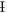

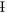
p < 0.01;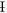 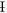 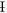
p < 0.001.

The pathologic findings of increased leukocyte trafficking and greater lesion pathology are suggestive of increased inflammation due to HIV in the setting of *Mtb* infection. To determine if a unique inflammatory response to pulmonary *Mtb* infection occurs in HuMice with HIV infection, we performed bioplex ELISA analysis of the lung supernatant from non-infected control animals, and animals infected with HIV, *Mtb*, and *Mtb* plus HIV. As shown in Fig. 6, the expression of most cytokines and chemokines in animals infected with only HIV were similar to the baseline presence observed in non-infected control animals (Fig. 6). The exceptions were a significant increase in MCP-1 and a trend towards increased IL-6 due to HIV infection. Consistent with the non-significant reduction of CD4^+^ T-cell populations that we observed at this stage of HIV infection (Fig. 1), there were no significant decreases in IFN-γ activation due to HIV co-infection with *Mtb*. As expected, several cytokines and chemokines were significantly increased in the lung microenvironment due to *Mtb* infection, including pro-inflammatory cytokines (IL-1-β, IL-6, and TNF-α) and chemokines (MCP-1, IP-10, Eotaxin 1, and GM-CSF) and IL-12, IFN-γ, and IL-4.

An exacerbation of pro-inflammatory cytokine and chemokine production, however, was observed in the pulmonary micro-environment due to co-infection. Significantly increased production of IL-1β, IL-6, and IP-10 was observed in co-infected lung as compared to *Mtb*-infected lung. Significant increases in IL-8 and MIP-1α were observed in co-infected lung while these molecules were not increased following *Mtb* or HIV mono-infection. Activation of IL-10 occurred following *Mtb* infection and was further increased due to co-infection, although this effect of co-infection failed to reach significance (p = 0.07) due to variability among animals.

### Pulmonary pneumonia and vascular occlusions develop as complications of HIV/*Mtb* co-infection

**Figure 7:**
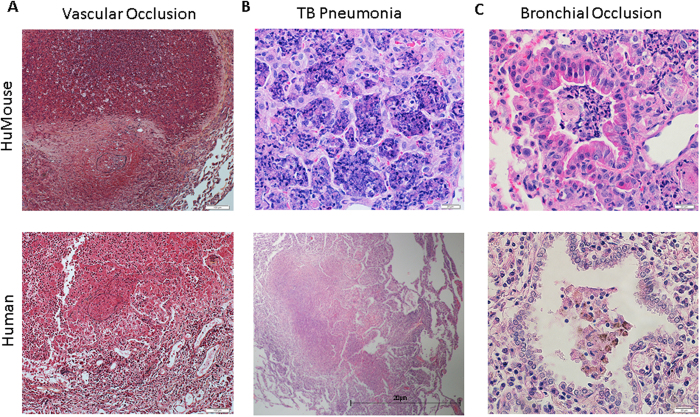
Pulmonary pneumonia and vascular occlusions develop as complications of HIV/*Mtb* co-infection. Visualization of lung pathology due to *Mtb* infection and *Mtb*/HIV co-infection in representative HuMice (top) and reference human (bottom) lung. (**A**) Movat staining reveals vascular occlusion as indicated by blockage interior to elastin bands of vessel (black). Visualization of H&E staining demonstrates (**B**) TB pneumonia and **(C)** bronchial occlusions in co-infected HuMouse (top) and human (bottom) lung.

Interestingly, TB pneumonia and vascular occlusions were noted in several animals with co-infection, while similar pathologies were not seen in in mono-infections. As shown in Fig. 7, alveolar spaces in co-infected HuMouse lung contained inflammatory cells and exudate consistent with TB pneumonia identified in infected human tissues. The development of TB pneumonia in immunocompromised persons including those with HIV/AIDS has been described^22^. Observation of this feature is an important endpoint that supports the validity of the HuMouse model to study TB/HIV. Additionally, infiltration of small vessels by mononuclear cells with endothelialitis and luminal narrowing in co-infected HuMouse lung was also observed. To confirm this finding, a pentachrome stain (Movat) was employed which identifies nuclei, elastin, cartilage, reticular fibers, mucin, fibrin, and muscle. Visualization of the black elastin fibers definitively identified vascular occlusions as shown within a representative tissue in Fig. 7A. Similar vascular occlusions were identified in lung from our co-infected human lung reference tissues as illustrated in Fig. 7B. Development of vascular occlusions is a pathology that has previously been noted in advanced stages of human TB^34^,^35^,^36^,^37^,^38^, but has not been associated with HIV to our knowledge. Development of bronchiole obstructions in the lung of co-infected mice (Fig. 7C) was observed. Bronchiole obstructions can occur in advanced TB in human subjects^39^,^40^ and we have previously described development of this pathology in HuMice at later stages (>7 wk) of *Mtb* infection^20^. At earlier stages of infection (5 wk) in the HuMouse, we observed in two independent studies that bronchiole obstructions developed only in the lungs of co-infected animals.

## Discussion

Evidence-based studies that can inform development of novel countermeasures to reduce and treat TB/HIV are critically needed. Our description of dual disease here is the first, to our knowledge, to demonstrate the application of HuMice in understanding the pathogenesis of TB/HIV, or of any viral and bacterial co-infection. In the absence of *Mtb*, infection with HIV in HuMice led to systemic viral propagation and random distribution of HIV + cells to the lung interstitium. In contrast, a definitive localization of HIV + cells to the periphery of TB granulomas was observed in HuMouse and human lung as previously described in a rhesus macaque model of SIV/TB^11^. These are important observations as a small number of HIV-infected cells can exert local immune modulatory activity through the effects of cytokines (e.g IL-10) and potentially toxic HIV proteins (e.g. gp120, tat, nef)^41^,^42^. These observations provide evidence to support the theory that the inflammatory response to *Mtb* infection may recruit HIV + cells to the granuloma, which in turn disrupts local protective innate and acquired immune function required at this critical site for containment of *Mtb* in the host lung.

HIV infection in HuMice markedly altered the host immune containment of pulmonary *Mtb* infection similar to the accelerated progression of TB disease in people with HIV^25^. Infection with HIV may be disrupting innate immune processes involved in bacterial killing or antigen presention by macrophages as demonstrated using *ex vivo* analysis of human cells^43^,^44^,^45^ or by impairing T cell helper and/or cytotoxic activity that restricts *Mtb* growth in macrophages^4^,^46^,^47^,^48^. In our model of co-infection, we observed *in vivo* effects prior to significant loss of CD4^+^ T cells in peripheral blood; the latter is a clinical indicator of generalized immune suppression in human subjects with HIV^47^,^49^. At this early stage of infection, we did not observe increased HIV replication when assessing peripheral and splenic viral loads as has been observed in the BAL fluid of some human subjects with active TB^50^. Consistent with the limited observations of co-infected human lung^13^,^14^ we found that disorganized *Mtb* lesions develop in the setting of HIV infection in the HuMouse. Our results, however, demonstrate that diffuse and poorly organized *Mtb* lesions can develop from the onset of acute co-infection. This is important, as the effect of HIV to compromise TB host defense is postulated to be due to HIV-mediated deterioration of the already developed protective granuloma structure, leading to bacterial escape and dissemination^9^. That scenario is most likely to apply to patients with latent TB, or a resolving TB infections, who later become infected with HIV. In those with an active HIV infection who subsequently are infected with *Mtb*, the initial results in the HuMouse model imply that *de novo* formation of the granuloma is compromised and perhaps occurs prior to lowering of CD4^+^ T cell counts in peripheral blood.

A surprising finding is that inappropriate or dysregulated immune responses, as opposed to immune suppression, can occur at an early stage of co-infection. It is important to note that the current studies were not extended to include late stages of disease characterized by CD4 + T cell loss and AIDS. In support of an earlier morphological description of cellular influx in a co-infected lung^14^, our analysis revealed neutrophils as the definitive inflammatory cell type accumulating in the lung of co-infected human subjects, and HuMice, at sites of *Mtb* proliferation. In the absence of HIV infection, neutrophils have a well-defined protective role during the early immune response to *Mtb*^32^,^51^ but are associated with pulmonary pathology when the infection fails to resolve^52^,^53^. The tissue destruction mediated by neutrophil degranulation and the accumulating debris from dying neutrophils provides an environment that is believed to be favorable for *Mtb* proliferation^21^. Our findings introduce a mechanistic concept that could explain why increased blood neutrophil counts in HIV co-infected patients is associated with greater TB disease severity^54^. Our studies have also suggested candidate inflammatory mediators (e.g. IL-1β, CXCL5, IL-8) that could promote excessive neutrophil recruitment in co-infected lung. Interestingly, these effects are observed in the absence of HIV-associated defects in several cytokines (IFN-γ, IL-12, and TFN-α) that play important roles in TB immunity^55^,^56^.

The development of vascular occlusions, bronchial obstructions, and bacterial pneumonia that we observed in co-infected animals and human reference tissue is consistent with the effects of exacerbated inflammation. Bacterial pneumonia is known to occur in people who are immune compromised who become infected with *Mtb*^22^, while vascular occlusions and bronchial obstructions are features observed in advanced TB disease^35^,^37^,^38^. We have previously reported development of bronchial obstructions and pneumonia in HuMice at later stages of *Mtb* infection (>7 wk p.i.) or following infection with a larger i.n. *Mtb* dose^20^. Our results in the HuMouse, then, suggest that HIV co-infection may accelerate the TB disease process by promoting specific aspects of the inflammatory process.

In summary, these studies describe a novel model that has been successfully applied to increase our understanding of TB/HIV co-infection pathobiology. These initial findings may provide a basis for the conflicting evidence for both immune activation and immune suppression in co-infected human subjects. Speculatively, co-infection may drive non-protective inflammation during early stages of HIV disease while immune compromise, including loss of IFN-γ, predominates once CD4^+^ T cell depletion ensues. In future studies it will be important to determine if these features of inflammatory pathology are promoted by the increased bacterial load in the HIV-compromised host, or if HIV-driven inflammation may play a more direct role in supporting the *Mtb* growth that subsequently accelerates TB disease progression. This is quite intriguing given recent studies that demonstrate inflammatory processes may augment *M.tb* proliferation^57^,^58^ independent of the effect of tissue destruction to provide a favorable environment for *Mtb* growth. Long term, the HuMouse may serve as an important pre-clinical model to identify mechanisms of co-infection pathobiology and test therapeutic interventions prior to use in NHPs or human subjects.

## Materials and Methods

### Ethics statement

All animal procedures were performed in accordance with the regulations of the NIH Office of Laboratory Animal Welfare and were approved by the University of Texas Medical Branch (UTMB) Institutional Animal Care and Use Committee (IACUC). Human fetal tissue used in generation of humanized mice was obtained via a non-profit partner (Advanced Bioscience Resources, Alameda, CA) as approved under exemption 4 in the HHS regulations (45 CFR Part 46) as previously described^20^.

### Animal Experiments

Humanized BLT mice were generated as we have previously described^20^. HIV (JR-CSF strain) was obtained from the Baylor College of Medicine Center for AIDS Research Virology Core and stored at −80 **°**C until use. The JR-CSF strain is a human clinical HIV-1 isolate that is classically defined as M-tropic and infects both monocyte/macrophage and CD4^+^ T cells. HIV was diluted in PBS and mice were infected with a 2,500 TCID_50_ via tail vein infection as described^17^. Infection with the H37Rv (tdTomato) strain of *Mtb*^59^ was performed using 250 CFU diluted in 40 ul of Dulbecco’s Phosphate-Buffered Saline (PBS, Cellgro, Manassas, VA, USA) via an intranasal route (20 ul/nare) as described^20^. Animal experiments were repeated in an independent group generated from an additional human tissue donor. Humanized mice are challenging to generate and animal numbers were a limitation. Two independent studies with non-infected animals (n = 2/study), *Mtb*-infected (n = 3/study) and *Mtb*/HIV-infected (n = 4/study) were performed. Three HIV-infected control animals were included only in one study. All animal experiments and work with *Mtb* were performed in a CDC-approved animal biological safety level-3 (ABSL-3) and BSL3 facilities in the Galveston National Laboratory in accordance with biosafety procedures approved by the UTMB Environmental Health and Safety Division.

### Bacterial and Viral Load

Following aseptic removal at 5 wk post *Mtb* infection, organs (lung and liver) were placed into 1 ml of PBS in 15 ml small tissue grinders (Kendall, Mansfield, MA, USA) for tissue homogenization. Serial dilutions of homogenized organ samples were prepared in PBS to perform limiting dilution CFU enumeration as described^60^. All studies were performed in a CDC-approved biological safety level-3 (BSL-3) facility. For viral quantification, plasma and supernatants from disrupted spleen were stored frozen at −80 °C. Plasma and splenic supernatants were then used to detect HIV p24 capsid protein using a clinical ELISA test (Zeptometrix, Buffalo, NY). Levels of p24 were quantified by generating a standard curve using the standards provided in the kit. Values were generated by linear regression to the standard curve as recommended by the manufacturer.

### Human Reference Tissue

Human lung tissue was obtained in collaboration with the UTMB Autopsy services from archival formalin-fixed paraffin embedded tissue obtained between 1996 and 2001. The reference samples included in Figs 1,4, and 7 are from decedents with confirmed TB or TB/HIV disease based on chart history and post-mortem analysis. Tissue were fixed in 10% formalin and embedded in paraffin until use to generate sections for analysis by histopathology and immunohistochemistry.

### Flow Cytometry

Levels of CD4^+^ T-cells were assessed pre- and post-infection by multi-variate flow cytometric assessment of peripheral blood. Blood was collected from the tail vein of HuMice into 300 μl of 3 mM ethylenediaminetetraacetic acid (EDTA, Capitol Scientific, Austin, TX, USA). Red Blood Cell Lysis Buffer (Sigma, St. Louis, MO, USA) was used according to the manufacturer to isolate the peripheral blood mononuclear cells (PBMCs). To reduce non-specific binding, PBMCs were incubated with CD16/CD32 Fc Block (BD Biosciences, San Jose, CA, USA). The following antibodies specific to human lymphocyte surface markers were used to stain the cells: AmCyan-CD45, APC-Cy7-CD3, and Pacific Blue-CD4. A total of 50,000 gated events were collected in a live cell gate using a BD LSR II (Fortessa) flow cytometer (BD Biosciences) in the UTMB Flow Cytometry and Cell Sorting Core Facility. Analysis of data was performed by FCS Express (*De Novo*, Los Angeles, CA, USA) software. Isotype matched antibodies were used to control for background fluorescence. Cells were selected based on expression of the human CD45 marker and further analyzed for human CD3 and CD4 phenotype.

### Immunohistochemistry

Lung tissue sections from formalin-fixed, paraffin embedded HuMouse or human lung were deparaffinized and washed in 0.05% PBST. Following deparaffinization, antigen retrieval was performed by 25 min incubation in citrate buffer, pH 6 (Dako) at 95–100 °C in a rice steamer. Samples were then washed with 0.05% PBST and incubated with monoclonal antibodies to MPO (at a dilution of 1:50; Abcam; ref ab45977-100) for two hours at room temperature or HIVp24 (at a dilution of 1:50; Dako; clone Kal-1) overnight at 4 °C. Sections were stained with Permanent Red using the Dako EnVision System with Mayer’s Hematoxylin as the counter stain. Images were taken using an Olympus BX53 microscope equipped with an Olympus DP71 camera.

### Pathology

At specified time points, following aseptic removal of organs, the remaining half of tissues were placed in 10% Neutral Buffered Formalin (Statlab, McKinney, TX, USA) for 48 hours to inactivate infectious agent, changed after 24 hours, and finally stored in 70% ethanol. Tissues were embedded in paraffin then stained with hematoxylin and eosin (H&E) and additional sections were stained using the Ziehl-Neelson method to visualize acid-fact bacteria (AFB) to detect *M.tb* bacilli. A 5 color chromagen (Movats) stain was performed to visualize vascular pathology including elastin, collagen, fibrin, mucin, and muscle fibers. Processing and staining for H&E, AFB, and Movats was performed by the University of Texas Medical Branch, Research Histopathology Core Facility. Lung tissue was evaluated by a trained pathologist with expertise in tuberculosis disease progression, with confirmation done in a slide blinded manner. Granuloma number was quantified from the entire section of mouse lung. The average size of each granuloma and the total amount of granulomatous tissue in the whole lung was quantified using digital software (NIH Image J; developed at the U.S. National Institutes of Health and available on the Internet at http://rsb.info.nih.gov/nih-image/). The percent of granulomatous tissue was calculated as a percent of total lung area as described^61^.

### Cytokine/Chemokine Quantification

Supernatants from disrupted lung were harvested during necropsy 5 wk post-*M.tb* infection and stored frozen at −80 °C. Samples were subsequently γ-irradiated on dry ice using a JL Shepherd Model 109–68 Cobalt-60 Research Irradiator (JL Shepherd & Associates, San Fernando, CA 91340) until 5 MRAD of exposure was reached. A non-diluted test sample was plated on 7H11 agar and incubated at 37 °C for >5 weeks to ensure sterility prior to removing samples from biocontainment. Lung supernatants were used in performance of a multiplex ELISA (Bio-rad Bio-plex Pro™ human cytokine 27-plex kit) according to the manufacturer’s instructions to assess changes in expression of human cytokines: IL-1β, IL-1ra, IL-2, IL-4, IL-5, IL-6, IL-7, IL-8, IL-9, IL-10, IL-12 (p70), IL-13, IL-15, IL-17, IP-10, FGF basic, Eotaxin, G-CSF, GM-CSF, IFN-γ, MCP-1 (MCAF), MIP-1α, MIP-1β, PDGF-BB, RANTES, TNF-α, VEGF. Values for cytokines where cross reactivity between human detection reagents and mouse cytokines could confound data (e.g. VEGF, IL-13) were excluded from the analysis. Levels of each cytokine were quantified by generating a standard curve using the standards provided in the kit. Values were generated by linear regression to the standard curve as recommended by the manufacturer and as described^62^. Values that were below the extrapolated range (<OOR) were set to 0.

### Statistics

Data are shown as mean ± SEM. One-way ANOVA followed by a Dunnetts multiple comparison test was used for multiple group comparisons (GraphPad Software v5.0). Statistically significant values are designated as follows: *p < 0.05; **p < 0.01; ***p < 0.001. The strength of association between viral load and bacterial burden or lung pathology was computed using the Spearman correlation coefficient (GraphPad Software v5.0).

## Additional Information

**How to cite this article**: Nusbaum, R. J. *et al*. Pulmonary Tuberculosis in Humanized Mice Infected with Hiv-1. *Sci. Rep.* **6**, 21522; doi: 10.1038/srep21522 (2016).

## Supplementary Material

Supplementary Information

## References

[b1] Organization, W. H. (World Health Organization, Geneva, 2014).

[b2] Harries A. D., Lawn S. D., Getahun H., Zachariah R. & Havlir D. V. HIV and tuberculosis–science and implementation to turn the tide and reduce deaths. J Int AIDS Soc 15, 17396 (2012). 22905358 10.7448/IAS.15.2.17396 PMC3499795

[b3] Khan F. A. . Treatment of active tuberculosis in HIV-coinfected patients: a systematic review and meta-analysis. Clin Infect Dis 50, 1288–99 (2010). 20353364 10.1086/651686

[b4] Law K. F., Jagirdar J., Weiden M. D., Bodkin M. & Rom W. N. Tuberculosis in HIV-positive patients: cellular response and immune activation in the lung. Am J Respir Crit Care Med 153, 1377–84 (1996). 8616569 10.1164/ajrccm.153.4.8616569

[b5] Markowitz N. . Incidence of tuberculosis in the United States among HIV-infected persons. The Pulmonary Complications of HIV Infection Study Group. Ann Intern Med 126, 123–32 (1997). 9005746 10.7326/0003-4819-126-2-199701150-00005

[b6] Mukadi Y. . Spectrum of immunodeficiency in HIV-1-infected patients with pulmonary tuberculosis in Zaire. Lancet 342, 143–6 (1993). 8101257 10.1016/0140-6736(93)91346-n

[b7] Naidoo K., Baxter C. & Abdool Karim S. S. When to start antiretroviral therapy during tuberculosis treatment? Curr Opin Infect Dis 26, 35–42 (2013). 23188213 10.1097/QCO.0b013e32835ba8f9 PMC3616247

[b8] McShane H. Co-infection with HIV and TB: double trouble. Int J STD AIDS 16, 95–100; quiz 101 (2005). 15807935 10.1258/0956462053057576

[b9] Diedrich C. R. & Flynn J. L. HIV-1/mycobacterium tuberculosis coinfection immunology: how does HIV-1 exacerbate tuberculosis? Infect Immun 79, 1407–17 (2011). 21245275 10.1128/IAI.01126-10 PMC3067569

[b10] Diedrich C. R. . Reactivation of latent tuberculosis in cynomolgus macaques infected with SIV is associated with early peripheral T cell depletion and not virus load. PLoS One 5, e9611 (2010). 20224771 10.1371/journal.pone.0009611 PMC2835744

[b11] Mehra S. . Reactivation of latent tuberculosis in rhesus macaques by coinfection with simian immunodeficiency virus. J Med Primatol 40, 233–43 (2011). 21781131 10.1111/j.1600-0684.2011.00485.x PMC3227019

[b12] Hanson D. L., Chu S. Y., Farizo K. M. & Ward J. W. Distribution of CD4 + T lymphocytes at diagnosis of acquired immunodeficiency syndrome-defining and other human immunodeficiency virus-related illnesses. The Adult and Adolescent Spectrum of HIV Disease Project Group. Arch Intern Med 155, 1537–42 (1995). 7605156

[b13] Bezuidenhout J., Roberts T., Muller L., van Helden P. & Walzl G. Pleural tuberculosis in patients with early HIV infection is associated with increased TNF-alpha expression and necrosis in granulomas. PLoS One 4, e4228 (2009). 19156215 10.1371/journal.pone.0004228 PMC2626629

[b14] de Noronha A. L., Bafica A., Nogueira L., Barral A. & Barral-Netto M. Lung granulomas from Mycobacterium tuberculosis/HIV-1 co-infected patients display decreased *in situ* TNF production. Pathol Res Pract 204, 155–61 (2008). 18096327 10.1016/j.prp.2007.10.008

[b15] Plaza V. . Bronchoalveolar lavage cell analysis in patients with human immunodeficiency virus related diseases. Thorax 44, 289–91 (1989). 2788319 10.1136/thx.44.4.289 PMC461795

[b16] Brainard D. M. . Induction of robust cellular and humoral virus-specific adaptive immune responses in human immunodeficiency virus-infected humanized BLT mice. J Virol 83, 7305–21 (2009). 19420076 10.1128/JVI.02207-08 PMC2704767

[b17] Choudhary S. K. . Latent HIV-1 infection of resting CD4(+) T cells in the humanized Rag2(-)/(-) gammac(-)/(-) mouse. J Virol 86, 114–20 (2012). 22013038 10.1128/JVI.05590-11 PMC3255863

[b18] Dudek T. E. . Rapid evolution of HIV-1 to functional CD8(+) T cell responses in humanized BLT mice. Sci Transl Med 4, 143ra98 (2012). 10.1126/scitranslmed.3003984 PMC3685142 22814851

[b19] Melkus M. W. . Humanized mice mount specific adaptive and innate immune responses to EBV and TSST-1. Nat Med 12, 1316–22 (2006). 17057712 10.1038/nm1431

[b20] Calderon V. E. . A humanized mouse model of tuberculosis. PLoS One 8, e63331 (2013). 23691024 10.1371/journal.pone.0063331 PMC3656943

[b21] Orme I. M. A new unifying theory of the pathogenesis of tuberculosis. Tuberculosis (Edinb) 94, 8–14 (2014). 24157189 10.1016/j.tube.2013.07.004 PMC3877201

[b22] Hunter R. L., Actor J. K., Hwang S. A., Karev V. & Jagannath C. Pathogenesis of post primary tuberculosis: immunity and hypersensitivity in the development of cavities. Ann Clin Lab Sci 44, 365–87 (2014). 25361920

[b23] Houben R. M. . Human immunodeficiency virus associated tuberculosis more often due to recent infection than reactivation of latent infection. Int J Tuberc Lung Dis 15, 24–31 (2011). 21276292

[b24] Crampin A. C. . Recurrent TB: relapse or reinfection? The effect of HIV in a general population cohort in Malawi. AIDS 24, 417–26 (2010). 20042847 10.1097/QAD.0b013e32832f51cf PMC2917772

[b25] Sonnenberg P. . HIV-1 and recurrence, relapse, and reinfection of tuberculosis after cure: a cohort study in South African mineworkers. Lancet 358, 1687–93 (2001). 11728545 10.1016/S0140-6736(01)06712-5

[b26] Berges B. K. & Rowan M. R. The utility of the new generation of humanized mice to study HIV-1 infection: transmission, prevention, pathogenesis, and treatment. Retrovirology 8, 65 (2011). 21835012 10.1186/1742-4690-8-65 PMC3170263

[b27] Sun Z. . Intrarectal transmission, systemic infection, and CD4 + T cell depletion in humanized mice infected with HIV-1. J Exp Med 204, 705–14 (2007). 17389241 10.1084/jem.20062411 PMC2118553

[b28] Orme I. M. Development of new vaccines and drugs for TB: limitations and potential strategic errors. Future Microbiol 6, 161–77 (2011). 21366417 10.2217/fmb.10.168 PMC3122326

[b29] Hoff D. R. . Location of intra- and extracellular M. tuberculosis populations in lungs of mice and guinea pigs during disease progression and after drug treatment. PLoS One 6, e17550 (2011). 21445321 10.1371/journal.pone.0017550 PMC3061964

[b30] Ridley D. S. & Ridley M. J. Rationale for the histological spectrum of tuberculosis. A basis for classification. Pathology 19, 186–92 (1987). 3453999 10.3109/00313028709077132

[b31] Mattila J. T. . Microenvironments in tuberculous granulomas are delineated by distinct populations of macrophage subsets and expression of nitric oxide synthase and arginase isoforms. J Immunol 191, 773–84 (2013). 23749634 10.4049/jimmunol.1300113 PMC3746594

[b32] Pedrosa J. . Neutrophils play a protective nonphagocytic role in systemic Mycobacterium tuberculosis infection of mice. Infect Immun 68, 577–83 (2000). 10639420 10.1128/iai.68.2.577-583.2000 PMC97179

[b33] Yang C. T. . Neutrophils exert protection in the early tuberculous granuloma by oxidative killing of mycobacteria phagocytosed from infected macrophages. Cell Host Microbe 12, 301–12 (2012). 22980327 10.1016/j.chom.2012.07.009 PMC3638950

[b34] Fullerton D. G. . Pulmonary tuberculosis presenting with central retinal vein occlusion. Br J Ophthalmol 91, 1714–5 (2007). 18024819 10.1136/bjo.2007.114777 PMC2095510

[b35] Elkeslassy A. . Dilatation of deep medullary veins in cortical venous occlusion due to focal tuberculous leptomeningitis. Neuroradiology 39, 705–7 (1997). 9351105 10.1007/s002340050490

[b36] Sheen-Chen S. M. . Computed tomography and angiography in hepatic tuberculosis mimicking liver tumor. Int J Tuberc Lung Dis 5, 876–8 (2001). 11573902

[b37] Equi A. . Pulmonary artery occlusion from tuberculous lymphadenopathy in a child. Pediatr Pulmonol 31, 311–3 (2001). 11288216 10.1002/ppul.1046

[b38] Takeuchi H. . Splenic vein occlusion secondary to tuberculous lymphadenitis at the splenic hilum: report of a case. Surg Today 30, 383–5 (2000). 10795875 10.1007/s005950050606

[b39] Collins J., Blankenbaker D. & Stern E. J. CT patterns of bronchiolar disease: what is “tree-in-bud”? AJR Am J Roentgenol 171, 365–70 (1998). 9694453 10.2214/ajr.171.2.9694453

[b40] Hunter R. L., Jagannath C. & Actor J. K. Pathology of postprimary tuberculosis in humans and mice: contradiction of long-held beliefs. Tuberculosis (Edinb) 87, 267–78 (2007). 17369095 10.1016/j.tube.2006.11.003

[b41] Ghiglione Y. & Turk G. Nef performance in macrophages: the master orchestrator of viral persistence and spread. Curr HIV Res 9, 505–13 (2011). 22103834 10.2174/157016211798842080

[b42] Li J. C., Yim H. C. & Lau A. S. Role of HIV-1 Tat in AIDS pathogenesis: its effects on cytokine dysregulation and contributions to the pathogenesis of opportunistic infection. AIDS 24, 1609–23 (2010). 20588103 10.1097/QAD.0b013e32833ac6a0

[b43] Imperiali F. G. . Increased Mycobacterium tuberculosis growth in HIV-1-infected human macrophages: role of tumour necrosis factor-alpha. Clin Exp Immunol 123, 435–42 (2001). 11298131 10.1046/j.1365-2249.2001.01481.x PMC1906017

[b44] Jambo K. C. . Small alveolar macrophages are infected preferentially by HIV and exhibit impaired phagocytic function. Mucosal Immunol 7, 1116–26 (2014). 24472847 10.1038/mi.2013.127 PMC4009066

[b45] Polyak S. . Impaired class II expression and antigen uptake in monocytic cells after HIV-1 infection. J Immunol 159, 2177–88 (1997). 9278305

[b46] Caccamo N. . Analysis of Mycobacterium tuberculosis-specific CD8 T-cells in patients with active tuberculosis and in individuals with latent infection. PLoS One 4, e5528 (2009). 19436760 10.1371/journal.pone.0005528 PMC2678250

[b47] Fahey J. L. . Prognostic significance of plasma markers of immune activation, HIV viral load and CD4 T-cell measurements. AIDS 12, 1581–90 (1998). 9764776 10.1097/00002030-199813000-00004

[b48] Ongaya A. . Mycobacterium tuberculosis-specific CD8 + T cell recall in convalescing TB subjects with HIV co-infection. Tuberculosis (Edinb) 93 Suppl, S60–5 (2013). 24388651 10.1016/S1472-9792(13)70012-X

[b49] Mellors J. W. . Plasma viral load and CD4 + lymphocytes as prognostic markers of HIV-1 infection. Ann Intern Med 126, 946–54 (1997). 9182471 10.7326/0003-4819-126-12-199706150-00003

[b50] Nakata K. . Mycobacterium tuberculosis enhances human immunodeficiency virus-1 replication in the lung. Am J Respir Crit Care Med 155, 996–1003 (1997). 9117038 10.1164/ajrccm.155.3.9117038

[b51] Seiler P. . Early granuloma formation after aerosol Mycobacterium tuberculosis infection is regulated by neutrophils via CXCR3-signaling chemokines. Eur J Immunol 33, 2676–86 (2003). 14515251 10.1002/eji.200323956

[b52] Marzo E. . Damaging role of neutrophilic infiltration in a mouse model of progressive tuberculosis. Tuberculosis (Edinb) 94, 55–64 (2014). 24291066 10.1016/j.tube.2013.09.004

[b53] Cardona P. J. . Towards a ‘human-like’ model of tuberculosis: intranasal inoculation of LPS induces intragranulomatous lung necrosis in mice infected aerogenically with Mycobacterium tuberculosis. Scand J Immunol 53, 65–71 (2001). 11169208 10.1046/j.1365-3083.2001.00842.x

[b54] Kerkhoff A. D., Wood R., Lowe D. M., Vogt M. & Lawn S. D. Blood neutrophil counts in HIV-infected patients with pulmonary tuberculosis: association with sputum mycobacterial load. PLoS One 8, e67956 (2013). 23874476 10.1371/journal.pone.0067956 PMC3706476

[b55] Cooper A. M. . Disseminated tuberculosis in interferon gamma gene-disrupted mice. J Exp Med 178, 2243–7 (1993). 8245795 10.1084/jem.178.6.2243 PMC2191280

[b56] Ottenhoff T. H., Kumararatne D. & Casanova J. L. Novel human immunodeficiencies reveal the essential role of type-I cytokines in immunity to intracellular bacteria. Immunol Today 19, 491–4 (1998). 9818540 10.1016/s0167-5699(98)01321-8

[b57] Tobin D. M., Roca F. J., Ray J. P., Ko D. C. & Ramakrishnan L. An enzyme that inactivates the inflammatory mediator leukotriene b4 restricts mycobacterial infection. PLoS One 8, e67828 (2013). 23874453 10.1371/journal.pone.0067828 PMC3708926

[b58] Vilaplana C. . Ibuprofen therapy resulted in significantly decreased tissue bacillary loads and increased survival in a new murine experimental model of active tuberculosis. J Infect Dis 208, 199–202 (2013). 23564636 10.1093/infdis/jit152

[b59] Kong Y. . Whole-body imaging of infection using fluorescence. Curr Protoc Microbiol Chapter 2, Unit 2C 3 (2011). 21538304 10.1002/9780471729259.mc02c03s21

[b60] Endsley J. J. . Mycobacterium bovis BCG vaccination induces memory CD4 + T cells characterized by effector biomarker expression and anti-mycobacterial activity. Vaccine 25, 8384–94 (2007). 17996992 10.1016/j.vaccine.2007.10.011

[b61] Hwang S. A., Wilk K., Kruzel M. L. & Actor J. K. A novel recombinant human lactoferrin augments the BCG vaccine and protects alveolar integrity upon infection with Mycobacterium tuberculosis in mice. Vaccine 27, 3026–34 (2009). 19428915 10.1016/j.vaccine.2009.03.036 PMC2680785

[b62] Massey S. . Comparative Burkholderia pseudomallei natural history virulence studies using an aerosol murine model of infection. Sci Rep 4, 4305 (2014). 24603493 10.1038/srep04305 PMC3945929

