# Supplementary material for: Pulmonary Tuberculosis in Humanized Mice Infected with HIV-1
Source: Sci Rep. 2016 Feb 24;6:21522. doi: 10.1038/srep21522 (PMC4808832; doi:10.1038/srep21522)
Supplement: Supplementary Information [file srep21522-s1.pdf]

PULMONARY TUBERCULOSIS IN HUMANIZED MICE INFECTED WITH HIV-1

Authors: Rebecca J. Nusbaum[1†], Veronica E. Calderon[2†], Matthew B. Huante[1†], Putri Sutjita[1], Sudhamathi Vijayakumar[1], Katrina L. Lancaster[1], Robert L. Hunter[3], Jeffrey K. Actor[3], Jeffrey D. Cirillo[4], Judith Aronson[1], Benjamin B. Gelman[1], Joshua G. Lisinicchia[1], Gustavo Valbuena[1*], and Janice J. Endsley[1*]

Affiliations:

[1]University of Texas Medical Branch, Galveston, TX 77555, USA.

[2]University of Texas El Paso, El Paso, TX 79902, USA.

[3]University of Texas-Houston Health Science Center, Houston, TX 77030, USA.

[4]Texas A&M Health Sciences Center, College Station, TX 77853, USA.

[†]These authors contributed equally to this work

*To whom correspondence should be addressed. E-mail: gvalbuen@utmb.edu, jjendsle@utmb.edu.

Fig. S1. Early cellular influx to the lung in the setting of co-infection. (A) Tissue pathology visualized by H&E staining shows that occasional foci of inflammation are found in both *Mtb*-infected and *Mtb*/HIV co-infected animals 3 wk p.i. with *Mtb.* Greater cellular influx at the sites of *Mtb*-driven inflammation is observed in the HIV co-infected HuMice. Detection of HIV p24 by IHC (DAB) from a matched tissue section in the lung of a representative co-infected HuMouse shows HIV+ cells are found in the early cellular influx at sites of *Mtb*-driven inflammation. **(B)** CFU enumeration of HuMouse lung tissue from a randomly selected subset (n=2 per group) of HIV+ or HIV- animals demonstrates similar mycobacterial burden at 3 wk p.i. with *Mtb*.

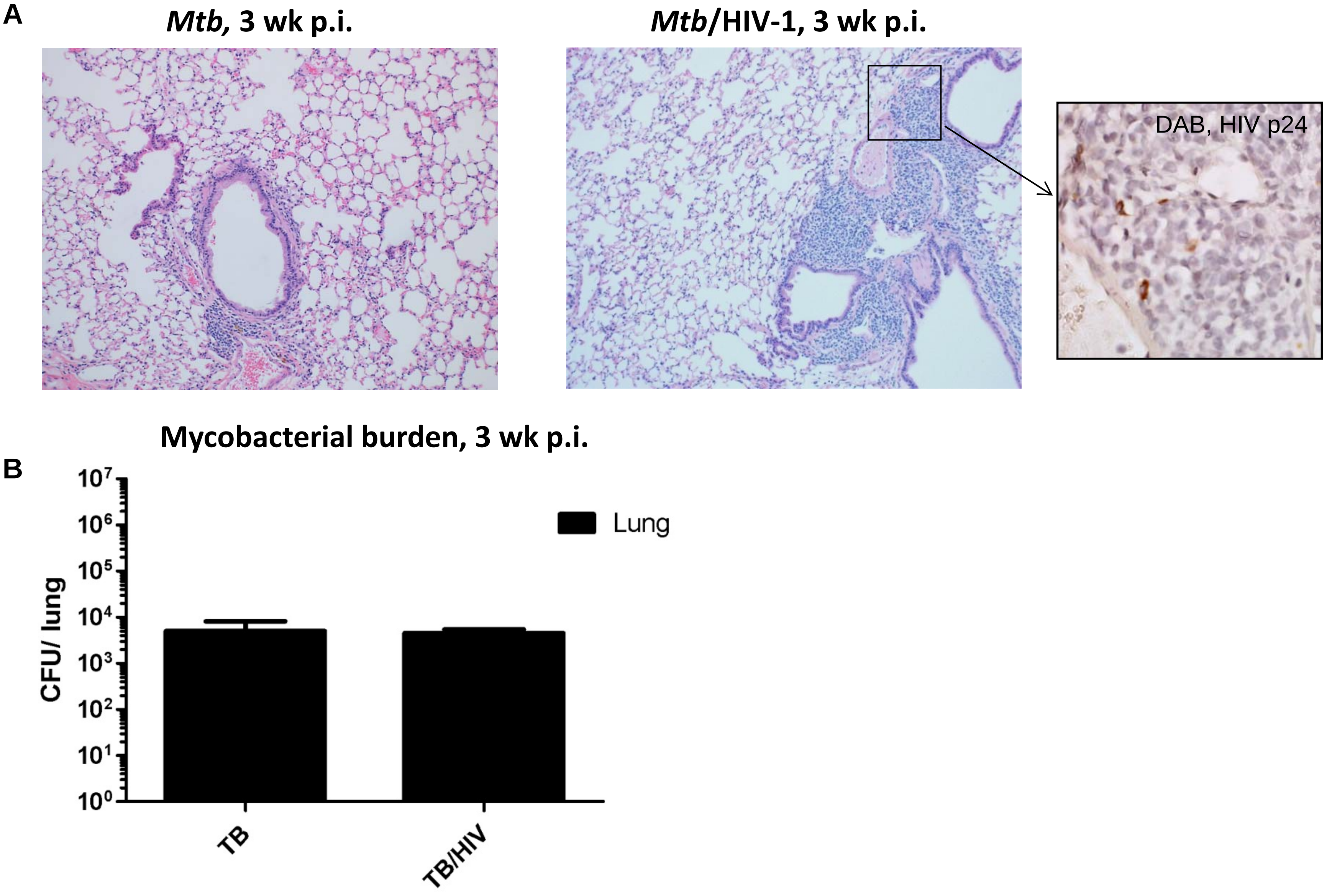
A
Mtb, 3 wk p.i.
Mtb/HIV-1, 3 wk p.i.
DAB, HIV p24
B
Mycobacterial burden, 3 wk p.i.
Lung
CFU/ lung
10^7
10^6
10^5
10^4
10^3
10^2
10^1
10^0
TB
TB/HIV
